# Implementation of an Integrated Sample Referral System (ISRS) in Ghana: Successes and Lessons Learnt from a Pilot Study in the Northern and Greater Accra Regions

**DOI:** 10.1371/journal.pgph.0004735

**Published:** 2025-09-11

**Authors:** Michael Owusu, Bernard Nkrumah, Godfred Acheampong, Stephen Opoku Afriyie, Ebenezer Kojo Addae, George Senyo Owusu, David Sambian, Joseph Asamoah Frimpong, Aliyu Mohammed, Abbas Abdul-Karim Komei, Gifty Boateng, Eunice Baiden Laryea, Pawan Angra, Farida Njelba Abdulai, Franklin Asiedu-Bekoe, Danielle T. Barradas

**Affiliations:** 1 Centre for Health System Strengthening, Kumasi, Ghana; 2 Department of Medical Diagnostics, Kwame Nkrumah University of Science and Technology, Kumasi, Ghana; 3 African Field Epidemiology Network, Accra, Ghana; 4 Centre for Remote Sensing and Geographic Information Services, University of Ghana, Legon, Accra, Ghana; 5 Department of Epidemiology and Biostatistics, School of Public Health, Kwame Nkrumah University of Science and Technology, Kumasi, Ghana; 6 Tamale Public Health Laboratory, Ghana Health Service, Tamale, Ghana; 7 National Public Health and Reference Laboratory, Ghana Health Service, Accra, Ghana; 8 Ghana Division of Global Health Protection, Global Health Center, U.S. Centers for Disease Control and Protection, Accra, Ghana; 9 Greater Accra Regional Health Directorate, Ghana Health Service, Accra, Ghana; 10 Public Health Division, Ghana Health Service, Accra, Ghana; PLOS: Public Library of Science, UNITED STATES OF AMERICA

## Abstract

The outbreak of the COVID-19 pandemic exposed the fragile health system in Ghana and in particular, its specimen referral system (SRS), which for years had been limited to Tuberculosis (TB), HIV Viral Load and Early Infant Diagnosis among few laboratories. The implementation of a siloed SRS with the sole focus on TB, HIV viral load, and early infant diagnosis has been to the detriment of other diseases of public health importance. This programmatic activity aimed to assess the feasibility of an integrated specimen referral system (ISRS) in Ghana’s Greater Accra and Northern Regions. Between January and November 2022, we established and piloted an ISRS through extensive stakeholder engagements using the hub and spoke model. As part of the intervention package, capacity building workshops, logistics support and transportation were provided to all program facilities. Standard paper-based log books and interviews were employed to collect data from 105 health facilities (84 in Greater Accra and 21 in Northern Region) to evaluate the ISRS’s successes, challenges, and develop future scale-up recommendations. Overall, 4,239 samples ranging from suspected cases of measles, monkeypox, COVID-19, yellow fever (YF), acute flaccid paralysis, TB, Buruli ulcer, HIV viral load (VL), early infant diagnosis (EID), and meningitis were transported during the program period. The turnaround time (TAT) varied across diseases: 1–2 days for TB/COVID-19, 5–7 days for YF, 7–14 days for influenza, and 14 days – 3 months for VL/EID cases. The quality of specimens referred improved, with only 2 samples rejected during the pilot. Qualitative analysis uncovered improvements in ISRS efficiency including, sample delivery, TAT, documentation, coordination and logistics, monitoring and tracking, sample quality, and record-keeping. This preliminary programmatic activity provides evidence of the feasibility of creating a nationwide ISRS to facilitate the quick detection and management of all diseases of public health importance in Ghana.

## Introduction

Laboratories play an important role in the early detection and reporting of infectious diseases. Activities of laboratories are most effective when organized into an integrated, multi-level network, facilitating timely access to appropriate diagnostic tools at each level [1,2]. The organisation of laboratories in an integrated system enables specimen to be referred from primary healthcare laboratories to higher-tiered laboratories that are equipped to perform diagnostic tests. A more efficient laboratory network for referral and transport of clinical and public health specimen enables rapid diagnosis, laboratory confirmation, and turn-around time of results, minimizing the time for reporting of new cases or an emerging outbreak and enhances safe and secure sample management [3–5].

The recent outbreak of SARS-CoV-2 in Ghana and many other sub-Saharan African countries exposed the fragile and fragmented specimen referral networks within the health system [6,7]. Even though Ghana has a wide network of both clinical and public health laboratories with good testing capacities, there is the lack of a robust network connecting these testing capacities. The lack of robust laboratory networks resulted in prolonged delays in the release of laboratory results and difficulty in accessing laboratory tests for patients from remote and rural areas. These deficiencies culminated in delays in the national response to the outbreak, resulting in delayed case management, risk communication, and other key aspects of the outbreak response [8].

Ghana has encountered several challenges with regards to the implementation of an efficient national integrated specimen referral system (ISRS). The implementation of a national specimen referral system from the lower-tiered health facilities to the referral laboratories is not well structured and is mainly driven by funding from the United States President’s Emergency Plan for AIDS Relief (PEPFAR) and the Global Fund [9] in support of tuberculosis (TB) and HIV viral load (VL) programmes. Within the current sample referral system in the Ghana Health Service (GHS), samples from suspected public health events are mainly transported to the laboratory through ad-hoc means, such as the use of public transport, personal vehicles, research-sponsored couriers, and government vehicles [9,10]. While some of these means were very expensive, the samples either arrived late to the laboratory, or in a condition that affected the integrity of the samples leading to prolonged turnaround times and low-quality results [10].

As part of addressing these challenges and leveraging on the currently existing testing capacities, in alignment with its Global Health Security Agenda, the United States Centers for Disease Control and Prevention in Ghana (CDC-Ghana) collaborated with the GHS to develop a comprehensive and holistic system for specimen referral that can support the efficient movement of samples for all diseases of public health importance [11]. CDC-Ghana funded the Centre for Health Systems Strengthening (CfHSS), a local health non-governmental organization, to pilot an integrated specimen referral system in two regions in Ghana. This programmatic activity gives an account of the processes involved and the feasibility of the ISRS implementation, as well as successes, challenges, and lessons learned. It explores the system’s impact on sample transportation efficiency, turnaround times, and overall laboratory network strengthening. Additionally, the programmatic activity provides insights into stakeholder engagement and resource allocation that support the ISRS. The findings will inform future scale-up efforts and contribute to optimizing specimen referral systems in similar settings.

## Materials and methods

### Project sites

The ISRS pilot was conducted in two regions of Ghana: the Greater Accra region (GAR) and the Northern region (NR). GAR is the most urbanized region in the country, with 91.7% of its total population living in urban centres [12]. The capital city of GAR is Accra, which doubles as the capital city of Ghana. The region is the most populated region, with over 5 million population [12]. GAR has 29 Districts, divided into Metropolitan, Municipal, and District Assemblies [13]. The region has 1,272 health facilities comprising 707 Community-based Health Planning and Services (CHPS) compounds, 299 clinics, 101 maternity homes, 32 health centres, 22 polyclinics, and 111 hospitals [13].

NR is located in the Northern part of Ghana, with a total population of 2.3 million [12] across its 16 districts, the capital of which is Tamale. The vegetation of NR is predominantly grassland and the main occupation in the region is agriculture. There are 314 health facilities in the region, comprising 32 hospitals, 2 polyclinics, 102 clinics/health centres, 7 maternity homes, and 171 CHPS compounds [14].

To test the system in different contexts, the GAR was selected due to its high population density, interlinked laboratories, and healthcare facilities, contrasting with NR, which was selected based on its lower population and sparsely distributed facilities.

### Ethical clearance

This program was performed through a collaboration between the United States Centers for Disease Control and Prevention and the Ghana Health Service as part of a systems strengthening approach to evaluate the sample referral systems in Ghana. We obtained a waiver from the Ethical Review Committee of the School of Medicine and Dentistry of the Kwame Nkrumah University of Science and Technology (CHRPE/AP/1176/24), which determined that the program posed minimal risk. The waiver approval permitted the use of anonymized data from our archives for further analysis and documentation. We also obtained a non-research determination from the United States Centers for Disease Control and Prevention (CGH-CSIB 7/17/20-97a75). Given the nature of the data and the waiver granted, obtaining individual participant consent was not required.

### Comprehensive participation in global research

Additional information regarding the ethical, cultural, and scientific considerations specific to comprehensive participation in global research has been added in the Supporting Information (S1 Checklist).

### Development process of the ISRS

**Fig 1:**
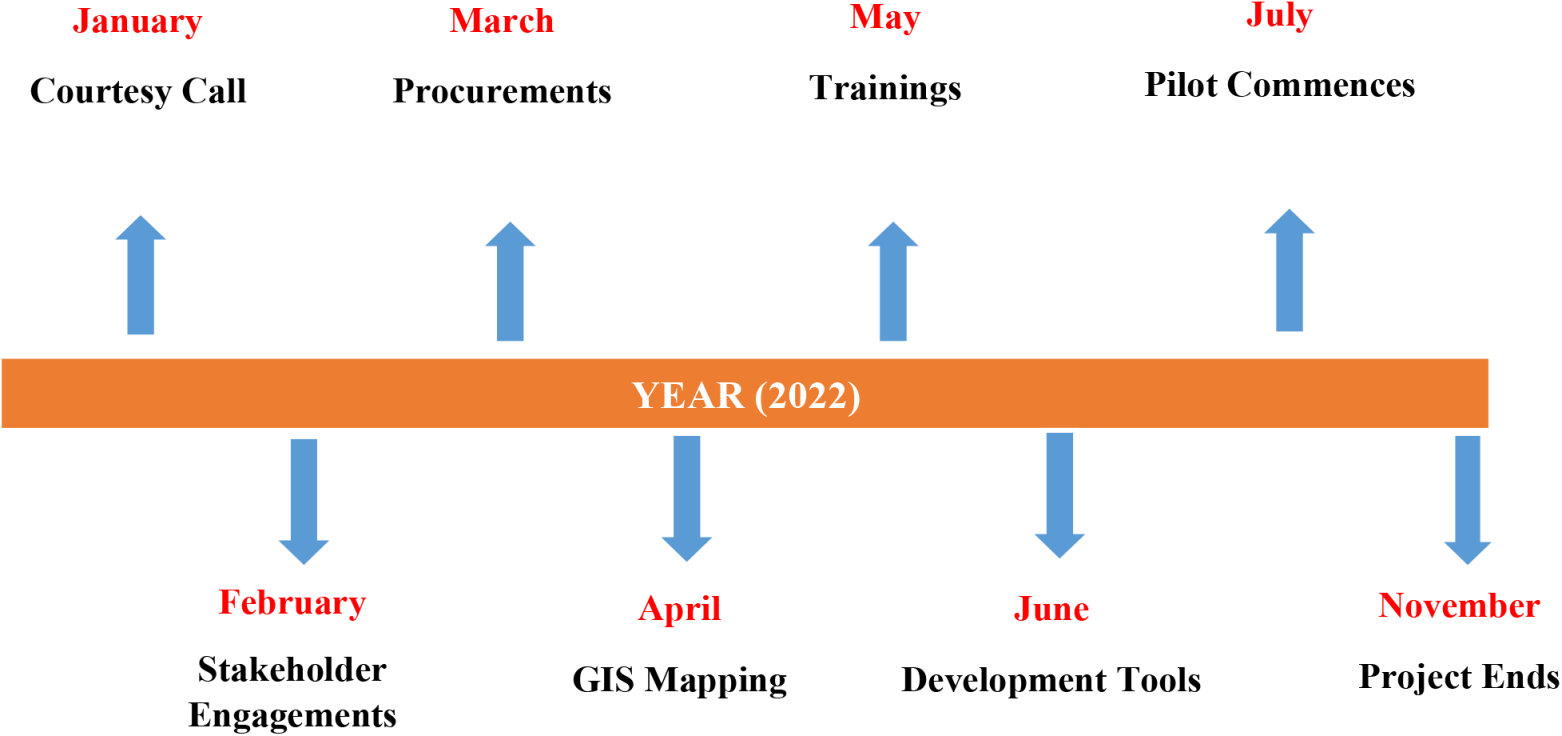
Timeline of activities of Sample Referral System pilot implementation in Ghana.

The planning and execution of the ISRS pilot, conducted from January through November 2022, employed a collaborative approach to ensure regional and district ownership of the process (Fig 1). This involved engaging regional and district health management teams, establishing technical working groups, and conducting extensive stakeholder consultations. To facilitate facility readiness, a baseline needs assessment was conducted to identify gaps in logistics and infrastructure. Where necessary, logistic support and transportation for samples were provided to facilitate smooth operations. Capacity building was a key component of the implementation, with targeted training sessions conducted for key stakeholders such as healthcare workers, drivers and motor riders. These sessions covered essential topics such as sample handling, biosafety and hazard management, and technical skills for transport personnel. The sample transportation model was designed through stakeholder consultations, ensuring alignment with existing local transportation options. A consensus was reached to leverage available transport options at the regional health directorates and private transporters: motorcycle riders were assigned to transport samples over short distances of ≤ 2 km from spokes to mini hubs whilst government-allocated vehicles were used to transport samples over long distances of more than 2 km from mini hubs to main hubs. The distance classifications were informed by factors such as regional preference, facility distribution, road infrastructure, and transport efficiency. In areas where healthcare facilities were clustered with bad road networks, motorcycles provided a fast and efficient means of transporting specimens from spokes to mini or main hubs.

Ensuring timeliness and maintaining sample integrity were also key considerations, as the classification helped optimize transport methods to prevent delays that could compromise specimen quality.

**Fig 2:**
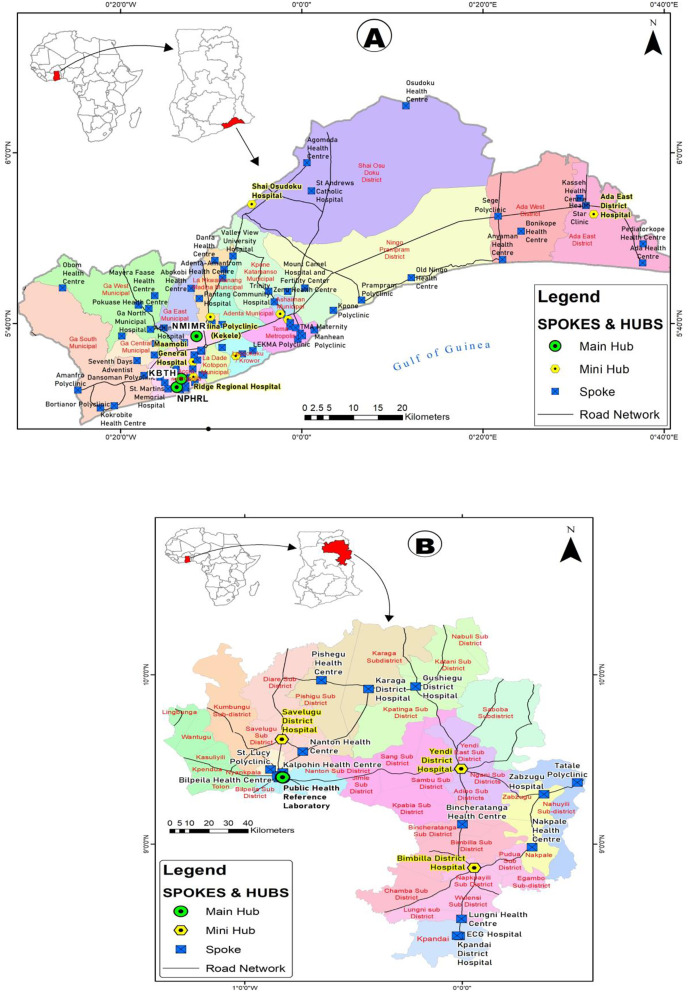
GIS maps showing the network of hubs and spokes in (A) Greater Accra Region and (B) Northern Region. ESRI ArcGIS 10.8 was used to produce the maps, while base layers were obtained from https://www.openstreetmap.org. The regional boundary shapefiles were sourced from https://drive.google.com/file/d/1AZ5PU3IH9CV_aZ9cmNDJEIfXJ2dQXrIQ/view?usp=drive_link and used in accordance with their licensing terms.

To ensure effective monitoring and evaluation (M&E), paper-based tracking tools were developed to measure system performance and address emerging challenges, with regular supervisory visits to provide oversight and support. Additionally, Geographic Information System (GIS) maps were developed to visualize the geographical distribution of health facilities and referral points, enhancing planning and decision-making (Fig 2). Overall, the ISRS pilot demonstrated a structured and integrated approach to strengthening specimen referral systems in both regions, with emphasis on stakeholder engagement, capacity building, data-driven decision-making and robust monitoring and evaluation mechanisms.

### Pilot implementation of integrated sample referral system

Trained GHS staff were designated as points of contact for specimen transport at each of the selected facilities. Motorcycles were used for specimen transport between spokes and mini hubs, while government vehicles were used for specimen movement from mini hubs to the main hubs/testing facilities. Sample transportation from spokes to hubs was done according to agreed scheduled times. During the pilot implementation, samples referred were categorized as either routine or on-demand. Routine samples were defined as samples that needed to be collected as part of investigations of patients who presented to the healthcare facilities or as part of on-going surveillance systems. On-demand samples were defined as samples that were collected during outbreaks at hotspots in the regions. Routine specimens were aggregated at the spokes temporarily (1–3 days) and sent twice per week to the mini hubs. Tuberculosis (TB) tests by GeneXpert were conducted at the mini hubs, and non-TB specimens were further transported (once a week) to the main hubs for testing. On-demand specimens were transported to the testing facilities immediately after collection. As part of this pilot activity, we provided cold boxes which had temperature monitoring logs for all sites involved in the programme. Temperature monitors were used to track the temperature of all samples during transportation and these were entered into the paper-based tracking tool used (S3 Appendix).

### Selection of districts and healthcare facilities

To optimize sample referral efficiency and to identify and select the testing and referral sites, the ISRS was designed by adapting the ‘hub and spoke’ model of specimen referral [4,15].

In consultation with the regional and district health directorates, healthcare facilities were selected for inclusion from each region based on the following criteria:

**Spokes:** Facilities that were part of the public health network involved in the collection of samples for diseases of public health importance, had the minimum capacity to package and transport samples according to national guidelines but did not have storage capacity.

**Mini Hubs:** Facilities with the capacity to collect (in some cases), process, package and store referred samples until they are transported to the main hub for testing. Some mini hubs also have the capacity to test for some diseases (e.g., TB and SARS-CoV-2 using GeneXpert). Mini hubs also served as a breakpoint between spokes and main hubs in instances where travel distances were longer than 2 km.

**Main Hubs:** In addition to meeting the spoke criteria, main hubs served as the referral centres capable of performing diagnostic testing for diseases of public health importance. Samples tested include HIV viral load, meningitis, yellow fever measles and other other suspected diseases in addition to TB GeneXpert and SARS-CoV-2.

**Minimum Distance of Travel:** Motorbikes were expected to travel within a 2 km radius. Vehicles were expected to travel above 2 km radius.

A total of 105 health facilities were selected in both regions: 84 from GAR (6.6%) and 21 from NR (6.7%). In GAR, the 84 facilities comprised 11 hubs (8 mini hubs and 3 main hubs) and 73 spokes, while NR facilities were composed of 4 hubs (3 mini hubs, 1 main hub) and 17 spokes. The 3 main hubs in GAR were the National Public Health and Reference Laboratory (NPHRL); the Central Laboratory, the Korle-Bu Teaching Hospital (KBTH); and the Noguchi Memorial Institute for Medical Research (NMIMR). In the Northern region, Tamale Public Health Laboratory was designated as the main hub (S1 Appendix).

### Programme design and data collection tools

This was a programme-based activity that involved qualitative and quantitative surveys using a data abstraction tool. Quantitative data were collected from specially designed laboratory logbooks, including sample reception books (S2 Appendix), sample transport vouchers (S3 Appendix), and test results logbooks (S4 Appendix). The logbooks were made available to all facilities selected to participate in the programmatic activity. Qualitative data were obtained from broad stakeholder consultations, observations, in-depth interviews, and focus group discussions (FGDs) before and after implementation of the programme. The interviews were conducted by public health experts and research assistants who were recruited as part of this programme. Key stakeholders engaged in the program included regional and district health directorate staff, disease surveillance officers, laboratory staff, drivers, and motor riders.

The qualitative component of the programme employed a mixed-methods approach to comprehensively evaluate stakeholder experiences and perceptions of the Integrated Sample Referral System implementation. Data collection involved 18 semi-structured interviews and 4 focus group discussions (FGDs) with key stakeholders directly involved in the sample referral processes. Participants included laboratory technicians (n = 12), laboratory managers (n = 8), courier service personnel (n = 6), and facility administrators (n = 4). The selection criteria ensured representation from both hub and spoke facilities, with participants having at least one year of experience with the laboratory referral system to provide meaningful pre-post implementation comparisons.

Interview and FGD protocols explored key domains of the sample referral system, focusing on transportation mechanisms, documentation processes, tracking systems, and reporting procedures before and after ISRS implementation. Each interview lasted approximately 45–60 min, while FGDs were conducted for 90–120 min. All sessions were audio-recorded and supplemented with field notes documenting non-verbal cues and contextual information.

The interview guide comprised four main sections: (1) pre-ISRS challenges in sample referral, including transportation delays, documentation inadequacies, and tracking limitations; (2) perceived post-implementation improvements in sample delivery speed, coordination, and documentation; (3) technical evaluations of new paper-based tracking tools; and (4) operational changes in reporting accuracy and turnaround times. The semi-structured format allowed for exploration of emerging topics while maintaining consistent core inquiries across all participants.

Additionally, hazard management training, defined as a structured program designed to educate healthcare and laboratory personnel on identifying, mitigating and responding to potential hazards related to sample collection, handling and transportation was conducted to ensure that field technicians, disease surveillance officers, motor riders and drivers involved in sample transportation adhered to biosafety protocols. To assess knowledge retention and training effectiveness, participants underwent pre- and post-training evaluations. These evaluations consisted of a 20-question assessment, including 10 practical multiple-choice questions (e.g., proper use of PPE, spill response protocols), 5 short-answer questions on hazard mitigation, and 5 scenario-based written questions testing theoretical knowledge (e.g., biosafety principles, hazard identification). The post-test was administered immediately after training and again three months after the start of the programme to evaluate long-term retention. To enhance system evaluation, key performance indicators such as turnaround time (TAT) and cost analysis were considered. We defined TAT as the duration between sample collection and result delivery to the requesting facility, serving as a crucial metric for assessing the efficiency of the referral system. Cost analysis, which translates as cost per kilometer, was determined by multiplying the total distance covered (in kilometers) by a standardized programme rate of $0.14/km. Total distance covered was calculated as the one-way distance between facilities multiplied by two (to account for round trips) and then multiplied by the total number of trips made. Kilometerage data was derived from GPS mapping, which took into account round-trip distances between health facilities (spoke or mini hub), and designated drop-off points (mini or main hub). These indicators were used to evaluate the financial sustainability of the ISRS, ensuring resources were allocated efficiently to improve diagnostic access while minimizing expenses.

### Data management and analysis

All data management procedures followed established protocols for ensuring data quality and confidentiality. Quantitative data, including sample referral volumes, turnaround times, and rejection rates, were entered and cleaned using Microsoft Excel 2019 (Microsoft, USA). These data were analyzed using descriptive statistical measures including frequencies, percentages, means, and standard deviations to quantify system performance metrics.

Qualitative data were collected through semi-structured open-ended questionnaires, audio recordings of in-depth interviews, and field notes from observations. All audio recordings were transcribed verbatim by two trained research assistants and translated into English where participants had communicated in local languages. To ensure translation accuracy, a third researcher conducted back-translation verification on a 20% random sample of the translated transcripts.

Thematic analysis followed a six-step process based on Braun and Clarke’s framework. First, two independent coders familiarized themselves with the data through repeated transcript readings. Second, initial codes were generated using an inductive approach, with intercoder reliability assessed through Cohen’s kappa coefficient (κ = 0.84). Third, preliminary themes were identified from the coded data segments. Fourth, themes were reviewed against the original dataset to ensure comprehensive representation of participant experiences. Fifth, themes were defined and named according to their conceptual focus. Finally, compelling extract examples were selected to illustrate each theme.

Data saturation was determined when three consecutive interviews yielded no new codes or thematic insights. The final analytical framework comprised six major themes: enhanced efficiency in sample delivery, improved turnaround time and documentation, enhanced monitoring and tracking, strengthened coordination and logistics, enhanced sample quality, and improved record-keeping. These themes provided a structured framework for comparing pre- and post-ISRS implementation phases. Member checking was conducted with five key participants to validate the accuracy of the thematic interpretations and enhance analytical rigor.

## Results

### Baseline challenges and interventions

Needs assessment of the selected health facilities revealed the lack of adequate sample referral logistics, documentation, and infrastructure at GAR and NR. The lack of dedicated couriers, poor road networks, long distances between certain referring laboratories, and cost of transportation were cited as challenges with regards to regular sample referral among laboratories.

To mitigate these logistical challenges, sample collection containers, biohazard bags, triple packaging materials, cryo-tubes, and cryo-boxes were procured for each region prior to the implementation of the ISRS pilot. Specially designed laboratory logbooks and specimen referral vouchers were also provided to the health facilities to ensure proper documentation. We coordinated with all transporters through the regional health directorates to cover transportation costs for motorbikes and vehicles used in the ISRS. Transportation support was allocated based on the distance travelled (km) by each driver or rider. The total cost incurred during the sample referral implementation has been detailed in Table 1. The estimated cost of transportation was $0.14/km, compared to a baseline cost estimate of $0.6/km [16].

**Table 1:** Overall transportation cost for GAR and NR.

Region	Facility	Total Cost (USD)			
		Month 1	Month 2	Month 3	Grand Total
Northern	Savelugu Municipal Hospital	17.5	113.8	79.8	
	Yendi Municipal Hospital	190.3	96.3	149.4	
	Bimbilla Municipal Hospital	141.5	129.1	266.6	
	Tamale Public Health Lab	103.2	57.6	8.8	
Total		**452.6**	**396.7**	**504.6**	**1353.8**
		**Month 1**	**Month 2**	**Month 3/4** ^*^	
Greater Accra	Ada East District Hospital	200	511.1	981.8	
	Tema	173.3	346.7	1,168	
	Ashaiman Polyclinic	61.1	138.9	368.7	
	Lekma Hospital	66.7	122.2	317.3	
	Greater Accra Regional Hospital	33.3	72.2	150	
	madina kekele	93.3	186.7	573.3	
	Shai-OsuDoku Hospital	200	377.8	1,572	
	Mamobi General Hospital	41.1	76.7	175.1	
Total		**868.9**	**1832.2**	**5306.2**	**8007.3**

*For Greater Accra, data for months 3 and 4 were aggregated due to the decision to extend sample collection by an additional month, hence costing for the two months was aggregated.

### Training in hazard management

A hazard management training was held at GAR and NR for a total of 42 individuals including field technicians, disease surveillance officers, and motor riders. Participants were trained in appropriate sample packaging, managing spillage during sample transportation, proper use of PPE, and laboratory safety, among many other topics. Pre- and post-tests of participant knowledge were conducted revealing a significant increase in the knowledge of safe specimen collection, packaging, storage, and transport (Table 2). These improvements were essential in reducing sample contamination, optimizing transportation conditions, and ensuring adherence to biosafety standards, all of which contributed to the overall success of the system.

**Table 2:** Pre-and post-hazard management training assessment.

Project site	No. of participants	Pre-Training Mean score (%)	Post Training Mean score (%)
GAR	21	49.3/64 (77.0)	55.3/64 (86.4)
NR	21	47.8/64 (74.7)	58.4/64 (91.2)
Total	42	48.5/64 (75.8)	57/64 (89.1)

### Measurable outcomes

#### Overall distribution of samples.

In total, 4,239 samples were transported across both regions throughout the implementation phase of the SRS (Table 3). Specimens collected and transported in GAR were almost 5 times more than those collected in NR.

**Table 3:** Overview of referred specimens in both regions.

Samples referred/received	GAR	NR
Overall samples received	3,483	756
Samples received at the main hubmain hubs	2,688	372
Samples received and processed at the mini hubs	795	384

### Weekly distribution of samples

**Fig 3:**
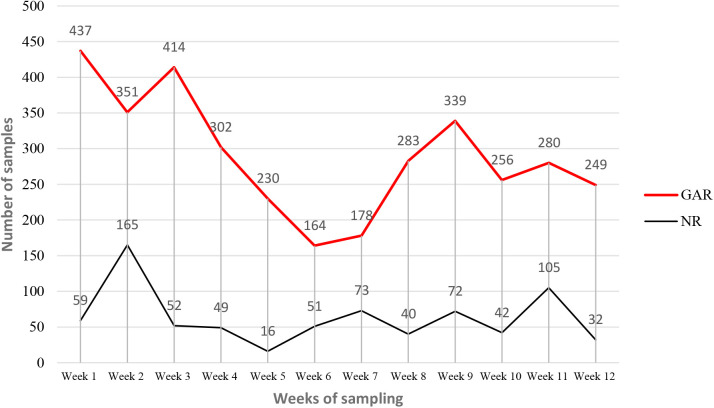
Weekly distribution of specimens referred to mini- and main hubs.

The weekly distribution of samples referred during the pilot implementation is summarized in Fig 3. In GAR, the highest number of referred samples were recorded in week 1 (n = 437, 12.5%) and the lowest in week 6 (n = 164, 4.7%). In NR, the highest number of referred samples were recorded in week 2 (n = 165, 21.8%) and the lowest (n = 16, 2.1%) in week 5.

### Monthly distribution of samples

**Fig 4:**
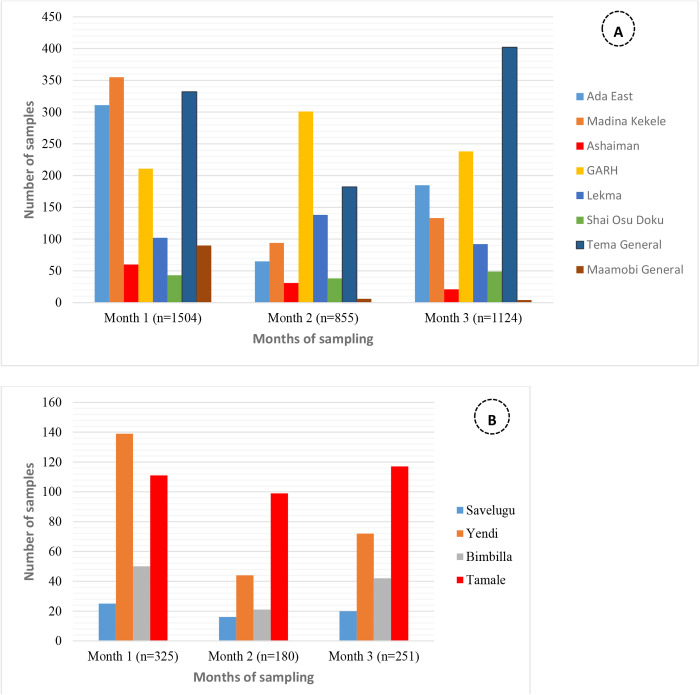
Monthly distribution of samples referred in (A) Greater Accra Region and (B) Northern Region by mini hub.

In both GAR and NR, the highest number of samples transported was observed in the first month of the pilot (Fig 4).

### Scope of specimens referred

In GAR, HIV viral load specimens (2,338; 67.1%) were the most collected and transported. However, TB specimens (517; 68.4%) were the most referred in NR. Certain suspected diseases were found only in GAR (Buruli ulcer and influenza) or only in NR (viral haemorrhagic fevers). Of the 4,239 specimens transported, 4,049 (95.5%) were routine samples. The total on-demand and routine samples in GAR were 146 and 3,337 respectively whilst NR recorded 44 on-demand and 712 routine samples. HIV viral load and HIV early infant diagnosis were done at KBTH (GAR), suspected yellow fever, acute flaccid paralysis and suspected measles were done at NPHR (GAR), Suspected viral haemorrhagic fever, suspected buruli ulcer and suspected influenza were done at NMIMR (GAR), Suspected meningitis were done at both NPHRL and TPHL (NR) and suspected COVID and Suspected tuberculosis were done at the mini hubs (Table 4).

**Table 4:** Scope of tests performed during implementation of the SRS pilot.

Suspected disease	Type of specimen	Sample type	GAR (N = 3,483) n (%)	NR (N = 756) n (%)
HIV viral load^*^	Plasma	Routine	2,338 (67.1)	168 (22.2)
HIV early infant diagnosis^*^	Plasma	Routine	204 (5.9)	27 (3.6)
Suspected tuberculosis$	Sputum	Routine	795 (22.8)	517 (68.4)
Suspected yellow fever^*^	Whole Blood	On-demand	2 (0.1)	8 (1.1)
Acute flaccid paralysis^*^	Stool	On-demand	3 (0.1)	6 (0.8)
Suspected COVID$	Oro- or nasopharyngeal swab	On-demand	86 (2.5)	4 (0.5)
Suspected measles^*^	Blood	On-demand	6 (0.2)	8 (1.1)
Suspected meningitis^*^	Cerebrospinal fluid (CSF)	On-demand	12 (0.3)	2 (0.3)
Suspected mpox^*^	Viral swab	On-demand	32 (0.9)	10 (1.3)
Suspected Buruli ulcer$	Wound swab	On-demand	2 (0.1)	0
Suspected influenza^*^	Nasopharyngeal swab	On-demand	3 (0.1)	0
Suspected viral hemorrhagic fever^*^	Blood	On-demand	0	6 (0.8)

*Tests conducted at main hubs.

$ Tests conducted at mini hubs.

### Performance indicators

Key performance indicators evaluated during the program have been summarized in Table 5. The average time for sample reception from spokes to the main hubs was 7 days in GAR (SD = 3.5, range = 3–11) and 4 (SD = 0.28, range = 3–4) in NR. At the mini hub level where TB tests were performed, average times for specimen reception and results release were greatly shortened. Only 2 samples were rejected during the pilot. The turnaround time, measured as the time duration between sample collection and reception of test results, differed across the selected facilities. The average TAT for samples received at the mini hub ranged from 24 hours to 3 days and those received at the main hub ranged from 10 to 14 days (Table 5). The TAT for specific suspected cases is described in Table 6. For both GAR and NR, suspected TB or COVID-19 cases recorded the shortest TAT (1–2 days) while HIV viral load and early infant diagnosis had the longest TAT (14 days to 3 months). Additionally, TAT for suspected cases of measles, meningitis, mpox and Buruli ulcer could not be estimated during the programmatic activity period for GAR due to delays from the reference public health laboratories.

**Table 5:** Performance indicators at the referral facilities (main- and mini hubs).

Indicator	Main Hubs		Mini Hubs	
	GAR	NR	GAR	NR
Total number of referred specimens received	2,688	372	795	384
Average time for specimens to be received at the referral laboratory	7 days (SD = 3.5, range = 3–11)	4 days (SD = 0.28, range = 3–4)	3 days (SD = 0.41, range = 3–4)	2 days (SD = 0.41, range = 2–3)
Total number of referred specimens received at the referral laboratory within the required time^*^	2,688 (100%)	370 (99.5%)	795 (100%)	384 (100%)
Number of specimens rejected due to inadequate or improper transport factors (e.g., hemolysis, spillage etc.)	0	1	0	0
Total number of referred specimens rejected for any other reason^**^	0	1	0	0
Average turnaround time for results release to spokes	10 days (SD = 0.91, range = 10–12)	14 days (SD = 0.33, range = 14–16)	3 days (SD = 3.0, range = 1–9)	24 hours
Total test results released within the laboratory’s specified turnaround time (TAT)	682 (25.4%)	138 (37.1%)	762 (95.8%)	384 (100%)

*required time is based on specimen type.

**other reasons for sample rejection: empty sample tube.

**Table 6:** Range of turn-around times per suspected case by region.

Suspected disease	GAR	NR
HIV viral load^*^	14 days – 3 months	14 days – 3 months
HIV early infant diagnosis^*^	14 days – 3 months	14 days – 3 months
tuberculosis	1-2 days	1-2 days
yellow fever	5-7 days	7-14 days
Acute flaccid paralysis	NE	NE
COVID-19	1-2 days	1-2 days
measles	NE	7-14 days
meningitis	NE	1-2 days
mpox	NE	7-14 days
Buruli ulcer	NE	NA
Influenza	7-14 days	NA
viral haemorrhagic fever	NA	7-14 days

*The equipment used for testing suffered a breakdown during the program period.

NE: TAT could not be estimated due to delays from the main hubs.

NA: No specimen collected for testing.

### Impact assessment of SRS pilot

The qualitative survey assessed six (6) major areas of the ISRS, as detailed in Table 7. The results revealed key thematic areas such as enhanced efficiency in Sample Delivery, improved Turnaround Time and Documentation, enhanced Monitoring and Tracking, strengthened Coordination and Logistics, and enhanced Sample Quality, and Record-Keeping.

**Table 7:** Qualitative impact assessment of the sample referral pilot implementation.

Indicator	Before SRS pilot	After SRS pilot
General overview of sample referral system	1. Delayed and irregular specimen transport 2. Difficulty in finding couriers 3. Cost constraints 4. Inadequate documentation of referred samples 5. Poor tracking of referred samples	1. Samples delivered at a faster rate 2. Shorter turnaround time 3. Detailed documentation for better reporting of results 4. Improved monitoring and tracking of referred specimens 5. Improved coordination between hubs and spokes
Sample transportation during SRS pilot vs. services provided by Ghana Post Courier Service	1. Delays and irregularities in sample pickup 2. Lack of dedicated couriers for specimen transport 3. Inadequate documentation 4. Poor tracking of samples referred 5. Concerns about sample integrity (e.g., spills)	1. Much more organized and regular delivery of samples 2. Samples delivered at faster rate with dedicated couriers 3. Detailed documentation for better reporting of results 4. Improved monitoring and tracking of referred samples 5. Improved sample integrity due to the provision of specialized sample referral containers and tracking systems
Logbooks for recording clinical information of referred samples	1. Logbooks for sample transport were not available 2. Exercise book with manually drawn lines were used for recording samples referred	1. Well categorized logbook for easy sorting and tabulation
Recording temperature of referred samples from spokes to hubs	This was not done	Sample containers with in-built thermometers were used to keep record of sample temperature
Specimen transportation system	1. Irregularly paced (once a week) 2. High specimen backlogs at main hubs 3. Concerns about sample integrity	1. Well organized and convenient sample delivery system 2. Improved delivery of samples reduced backlogs at main hubs 3. Improved monitoring and tracking of referred samples has improved sample quality
Reporting of results	1. Soft copies sent through WhatsApp platform to facilities but this was not coordinated by the region 2. Results went missing sometimes	1.Improved communication of results between laboratories through dedicated coordinators 2. Results now entered in logbooks for better record keeping

### Enhanced efficiency in sample delivery

Prior to ISRS implementation, sample delivery was characterized by irregular schedules and significant delays. Laboratory personnel reported substantial challenges with sample transportation. As one laboratory technician described, *“Before ISRS, we struggled with unpredictable delivery schedules. Sometimes samples would sit for days waiting for transport.”*

The introduction of ISRS brought systematic improvements through trained dedicated couriers and organized delivery schedules. The Sample Referral Coordinator, A medical superintendent noted the transformation: *“Now our sample delivery system runs like clockwork. We have regular pickup times, and samples reach the testing facilities much faster than before.”*

### Improved turnaround time and documentation

The pre-ISRS period was marked by lengthy turnaround times and inadequate documentation processes. *“Previously, it could take weeks to get results back, and tracking the progress was nearly impossible,”* recalled a laboratory supervisor. Post-implementation, significant improvements were observed in both areas. A laboratory coordinator emphasized, *“With ISRS, not only have we cut our turnaround times by more than half, but we can also document and track each step of the process clearly, thanks to the program’s distributed logbooks. This has greatly improved our service delivery and patient care.”*

### Enhanced monitoring and tracking

Monitoring and tracking capabilities were severely limited before ISRS implementation. Healthcare workers struggled with sample tracking and status updates. As one laboratory technologist stated, *“We had no reliable way to track integrated samples once they left our facility. “Few delegated logbooks were only used for the siloed programmes (HIV, TB, EID)”. It was like sending them into a black hole.”* The new system introduced comprehensive integrated tracking mechanisms. *“Now we can monitor samples at every stage of transport and processing. The tracking system gives us real-time updates, which has significantly reduced the number of lost or delayed samples,”* reported a hub facility manager.

### Strengthened coordination and logistics

The pre-ISRS period suffered from poor coordination between facilities and inadequate logistics management. A facility coordinator described the previous situation: *“Coordination between hubs and spokes was minimal, leading to frequent miscommunication and logistical challenges.”*

ISRS implementation brought structured coordination protocols and improved logistics management. *“The new system has created clear communication channels between facilities. We now have designated coordinators and established protocols for managing logistics,”* noted a senior laboratory administrator.

### Enhanced sample quality

Sample quality was a significant concern before ISRS implementation. Healthcare workers expressed anxiety about sample integrity during transport. *“We often received compromised samples due to improper handling and transportation conditions,”* recalled a laboratory technician. The introduction of specialized transport containers and quality control measures through ISRS has markedly improved sample integrity. A quality control supervisor noted,

*“The new transport containers and protocols have significantly reduced sample rejection rates”. We now maintain proper temperature control and handling conditions throughout the transport process.”*

### Record-keeping

The pre-ISRS record-keeping system was rudimentary, relying on manual entries in basic notebooks. *“Our previous record-keeping system was prone to errors and made it difficult to retrieve information when needed,”* stated a laboratory staff member. ISRS introduced standardized logbooks and systematic recording procedures. A facility manager highlighted the improvement: *“The new record-keeping system has transformed our data management. We can now easily access historical data, track trends, and generate reports for better decision-making on diseases of public health importance.”*

A summary of the key areas of discussion is shown below:

## Discussion

This programmatic activity reports on the implementation of an integrated sample referral system pilot in two regions in Ghana, highlighting the successes, challenges and lessons learnt for potential future scale-up. At present, there is no robust integrated specimen referral system to enhance prompt surveillance and response to a wide range of diseases of public health importance. Prior to the pilot, the majority of the respondents reported delays and irregularities in sample transportation and prolonged turnaround times. Most of these challenges were due to lack of dedicated couriers, not well-defined periods for collection and transport of samples, inadequate availability of specimen transport logistics such as biohazard bags, sample transport containers or coolers, and little to no training on sample handling and hazard management for healthcare staff and couriers.

The results of the ISRS pilot implementation in Ghana revealed several key findings with significant implications for policy and practice. The baseline assessment identified critical gaps in sample referral logistics, documentation, and infrastructure in both regions. To address these challenges, logistics support including sample collection containers, biohazard bags, and fuel reimbursements were provided, alongside specialized training in hazard management for healthcare staff. Similar studies in other low-resource settings have demonstrated that structured investments in transportation logistics and personnel training significantly improve sample integrity and reduce TAT, ultimately improving accuracy in diagnostics and disease surveillance [3,4,17].

This programmatic activity which assessed an integrated SRS represents a strategic shift from disease-specific vertical referral models to a cost effective, integrated framework that enhances both scalability and sustainability. Central to this integrated framework is the adoption of a per kilometre cost structure. Our programmatic activity revealed that the cost/km for the ISRS was cheaper ($0.14/km), a difference of $0.46/km, compared to a similar report conducted in Ghana by the Diagnostic Network Optimization [16]. In contrast to systems in African countries like that in Guinea and Ethiopia, where transport costs are calculated per specimen and tend to increase when specimen volumes are low, the ISRS offers a more economically viable model. For instance, Guinea reported an average cost of $7.50 per specimen irrespective of distance [15], while Ethiopia’s public-private partnership for specimen transport reflected high per-specimen costs up to $5.00 per specimen when few samples were batched per trip. In contrast, batching of 10 or more samples significantly reduced the cost to approximately $0.50 per specimen, illustrating the impact of volume on transport efficiency. This difference reflects notable efficiency gains and stresses the value of an integrated, distance-costing model. Moreover, the ISRS supports multiple public health programs such as HIV viral load and TB. This disease-integration avoids duplication of logistical efforts and promotes economies of scale. Shared infrastructure, unified training, and centralized quality control reduces costs across the broad advantages that vertical programs often fail to realize. Comparative studies in Zambia and Uganda have similarly shown that integrated systems yield better cost performance and service delivery [4,18].

The pilot programme successfully transported a total of 4,239 samples, within acceptable turn-around times across both regions. Prior to implementation, facilities engaged in this programme reported using public transport systems for referral and occasionally involved private couriers at exorbitant rates contributing to delays and inefficiencies [19,20]. Respondents from the main hubs reported having to work overtime to clear backlogs associated with delayed delivery of referred samples. Inadequate documentation and low-quality control of referred samples often led to poor tracking and poor sample integrity respectively. Spokes at remote areas particularly noted the poor road infrastructure and long spatial distance to existing referral laboratories hence leading to delays in turnaround time. Post-implementation, the ISRS demonstrated notable improvements. In both regions, specimen delivery to testing laboratories were generally completed within 3–4 days of collection. Although both regions operated under a decentralized model, return of test results was comparatively faster in Greater Accra (averaging 10 days) than in the Northern Region (averaging 14 days), reflecting differences in transport infrastructure, geographic coverage, and laboratory capacity. These findings mirror experiences from other decentralized models in resource-constraint settings. In Ethiopia for example, a public-private partnership for specimen referral led to a reduction in TAT from 7–10 days to 2–5 days [3]. Uganda similarly reported significant reductions in TAT for HIV-related diagnostics through its national laboratory sample transport system [4]. Research from Cameroon and Haiti also confirms that decentralized referral networks help reduce transportation barriers and improve access to timely diagnostics [5,21].

A critical aspect of this pilot is the impact assessment, which highlight s thematic areas for system enhancement, including improved coordination, monitoring, and tracking, as well as enhanced sample quality and record-keeping. A limitation of the current pilot however, is the predominantly qualitative approach to impact assessment. While user feedbacks provide crucial understanding of the system functionality, a more quantitative assessment of reduction in TATs, and overall diagnostic yield would offer stronger evidence of the ISRS’s benefits, as demonstrated by research in other LMICs [17,22]. That said, qualitative assessments remain essential, as they capture user experiences, contextual challenges, and operational barriers that quantitative data alone may overlook. Future studies may incorporate a holistic yet balanced mixed-method approach that could strengthen the case for the ISRS scale-up.

Sustained investment in the SRS is essential to maintain and scale-up the gains achieved through the ISRS. This investment should encompass not only financial resources but also policy reforms aimed at institutionalizing courier networks, optimizing sample transport schedules, and strengthening supportive supervision. Additionally, there is a need for further programmatic activities to systematically evaluate the long-term sustainability of the ISRS, particularly focusing on cost-efficiency, integration into national laboratory networks, and alignment with broader health system strategies. Strengthening specimen referral systems will be pivotal in enhancing Ghana’s capacity for timely disease detection, response, and overall public health preparedness. Embedding the ISRS within national health financing frameworks and leveraging multi-sectoral collaboration, including public-private partnerships, will be critical to reducing dependency on donor funding and ensuring continuity of operations in the face of evolving funding landscapes. These approaches align with global experiences, such as Ethiopia’s public-private partnership model, which successfully integrated specimen referral into routine national systems [3]. This improvement also has significant implications for policy, ensuring continued efficiency and effectiveness in healthcare service delivery.

Beyond diseases of public health importance, the ISRS framework enables expansion into bacteriology and antimicrobial susceptibility testing (AST). Ghana’s bloodstream infection (BSI) surveillance shows substantial rates of multidrug-resistant Gram-negative pathogens, including *Escherichia coli* and *Klebsiella pneumoniae*, signaling a growing need for AST services [23–25]. By leveraging the existing hub-and-spoke model, facilities without bacteriology capacity can refer specimens for culture and AST to regional or national laboratories to strengthen the country’s capacity to combat antimicrobial resistance, thereby supporting Ghana’s National Action Plan on Antimicrobial Use and Resistance [26]. Expanding bacteriology within ISRS would address critical diagnostic gaps by reducing delays in AST reporting, improving targeted antibiotic prescribing, and strengthening participation in global AMR surveillance initiatives [27]. Similar outcomes are documented in other settings: integrated specimen networks in Uganda and Haiti have improved diagnostic access and efficiency across multiple disease types [4,5], while Ethiopia’s partnership model demonstrated scalability beyond vertical disease transport [3]. By incorporating bacteriology into ISRS, Ghana can also enhance its One Health response to AMR. Integrated referral for culture and AST would enable better antibiogram generation, strengthen sentinel site capacity, and improve linkages between peripheral facilities and national reference laboratories [25]. In the long term, such expansion will be instrumental in building a resilient diagnostic network capable of addressing both infectious disease outbreaks and routine clinical needs.

The interpretation of the ISRS pilot findings must be situated within the context of certain data limitations and the short implementation timeframe. Firstly, the pilot was conducted over a limited duration, which restricts the ability to assess long-term sustainability, seasonal fluctuations in specimen flow, and systemic resilience under stress conditions. Consequently, while the results indicate clear improvements in cost-efficiency and turnaround time, these outcomes may not fully represent the challenges likely to emerge during broader scale-up.

Therefore, while the pilot demonstrates feasibility and promise, these limitations necessitate cautious interpretation. Future implementation phases should be accompanied by robust monitoring frameworks, extended observation periods, and a stronger emphasis on data quality assurance to generate evidence that can more reliably guide national scale-up.

### Lessons learned

#### Interventions and successes.

The pilot implementation prioritized capacity-building efforts to address the multifaceted challenges within the specimen referral process, targeting key stakeholders such as disease surveillance officers, couriers, laboratory staff, and district health directors. Through meticulously organized workshops, significant gains in knowledge were observed among participants, as evidenced by the substantial increase in scores documented in Table 2. Moreover, the pilot expanded the scope of samples referred beyond the traditional focus on TB, HIV VL, and EID, encompassing a broader range of diseases with public health significance like measles, mpox, and meningitis.

To facilitate seamless sample transport, the facilities received essential logistics support including sample transport containers, biohazard bags, packaging materials, and thermometers to monitor sample temperature during transit, addressing a previously overlooked aspect of the process.

Standardized paper-based logbooks and dedicated couriers were also provided to streamline documentation, monitoring, and transportation, resulting in shorter turnaround times and improved sample transport frequency.

The program fostered enhanced communication channels between lower-tier and specialized laboratories, bolstering a robust quality assurance system to ensure the reliability of results shared among health facilities. A significant success of the program was the ability to streamline transportation cost using distance travelled rather than number of samples transported. By developing this cost-per-distance metric, we established a transparent and consistent framework for calculating transportation expenses. This standardization improved budget planning, ensured fair compensation for transporters, and enhanced overall efficiency in sample referral logistics.

The development of GIS maps marked a significant milestone, facilitating an inclusive approach to facility selection across diverse geographical zones even though the designated travel distance for motorbikes (2 km) was low and needs to be reviewed. The usefulness of the GIS maps may be expanded to help strengthen local capacity for disease surveillance and management of public health emergencies [3,15].

This initiative permitted the integration of remote laboratories into the referral system and laid the foundation for potential scalability to the national level. The GIS maps hold promise for strengthening local capacity for disease surveillance and management of public health emergencies, serving as valuable tools for fostering communication and collaboration among geographically dispersed laboratories. The SRS pilot approach was successful in both rural and urban settings emphasising the sustainability of this for the long term.

### Challenges and limitations

The actual pilot implementation took place for a relatively shorter period than anticipated, hence, the long-term effect of the pilot could not be assessed. This was in part due to the need for extensive consultations with multiple partners and stakeholders involved in the ISRS who were often engaged in other competing priorities. These consultations were, however, very critical to obtain inputs and buy-in from these stakeholders to ensure the successful execution of the program. The lack of quantitative data for TAT and the number of tests prevented the team from conducting a thorough analysis to assess the impact of this intervention. Similarly, comparative assessment of costs incurred before and after the pilot implementation could not be done due to the unavailability of empirical financial data from the sites. We therefore relied on qualitative observations to assess the impact of this programme. Although qualitative assessment may have inherent weaknesses, we believe including this aspect could give an idea about the potential improvement in this intervention. The TAT reported for HIV Viral Load and EID were impacted by frequent shortages of reagents and breakdown of equipment. These could explain why the TAT were high. Although the overall estimated cost for our approach appears cheaper, we are unable to disaggregate based on the transport mode.

### Key takeaways

SRS implementation has been largely donor and disease driven. Various partners such as United States Centers for Disease Control and Prevention, the Global Fund, United States Agency for International Development, Korean International Cooperation Agency, World Health Organization, and others have supported SRS in different forms in the past and are continually supporting the system. To sustain SRS in Ghana, there is a need to coordinate and channel partner funding to augment the Ghana government’s contributions. Another consideration is the need for public-private participation where the private sector could help in accomplishing various tasks for courier/transportation, telecommunication, oil and gas, and information technology companies. There is the need to review and revise the 2 km travel distance for motorbikes from spokes to mini hubs to limit the use of vehicles and possibly make the ISRS implementation less expensive. To ensure a harmonized SRS implementation in Ghana, it is imperative that supportive policies and guidelines are developed, and healthcare workers and couriers are trained to understand and apply them in their day-to-day operations. At the minimum, the following key guidelines need to be developed: National Integrated Specimen Referral and Transport Network Guidelines, a National Guidelines on Sample Collection, Handling and Transportation using the One Health Approach and a National Biosafety and Biosecurity Guidelines.

## Supporting information

S1 Appendix Selected facilities for sample referral pilot studies. (DOCX)

S2 Appendix Specially designed laboratory sample reception logbook. (DOCX)

S3 Appendix Specially designed sample transport voucher. (DOCX)

S4 Appendix Specially designed test results logbook. (DOCX)

S1 Checklist Inclusivity in global research. (DOCX)
