# Supplementary material for: Implementation of an Integrated Sample Referral System (ISRS) in Ghana: Successes and Lessons Learnt from a Pilot Study in the Northern and Greater Accra Regions
Source: PLOS Glob Public Health. 2025 Sep 11;5(9):e0004735. doi: 10.1371/journal.pgph.0004735 (PMC12425209; doi:10.1371/journal.pgph.0004735)
Supplement: S1 Appendix — (DOCX) [file pgph.0004735.s001.docx]

| **SELECTED FACILITIES FOR SAMPLE REFERRAL PILOT PROGRAM** |
| --- |
| **GREATER ACCRA REGION** |

| **HUBS** | **Distance (Km)*** | **SPOKES** | **SUB-DISTRICT** |
| --- | --- | --- | --- |
| **ADA EAST DISTRICT HOSPITAL** | 7 | Kasseh Health Centre | Kasseh Sub District |
|  | 22 | Bornikope health centre | Bonikope Sub |
|  | 34 | Anyamam health centre | Anyamam |
|  | 19 | Pediatorkope Health Centre | Pediatorkope Sub District |
|  | 17 | Ada Health Centre | Ada Sub District |
|  | 17 | Sege Health Centre | Sege Sub District |
|  |  |  |  |
| **MADINA KEKELE** | 13 | St. John Of God Clinic | Koose |
|  | 8 | Ghana Canada Medical Centre | Sutsurunaa |
|  | 6 | Frafraha Health centre | Gbentanaa |
|  | 12 | Amanfrom Health centre | Koose |
|  | 11 | Amrahia Health centre | Gbentanaa |
|  | 4 | Ogbojo polyclinic | Nii Ashale |
|  | 5 | Nii Ashely health centre | Nii Ashale |
|  | 7 | Adjiringanore health centre | Sutsurunaa |
|  | 5 | Mother-love Hospital | Gbentanaa |
|  | 21 | Trinity Community Hospital | Mantseman |
|  | 4 | Adenta Clinic | Gbentanaa |
|  | 6 | Oyoko Clinic | Gbentanaa |
|  | 4 | Twumasiwaa Memorial Clinic | Sutsurunaa |
|  | 9 | Pantang Hospital | Pantang Sub-Municipal |
|  | 2 | Sanford World Clinic | Pantang Sub-Municipal |
|  | 17 | Danfa Health Centre | Danfa Sub Municipality |
|  | 2 | Madina Polyclinic (Rawlings Circle) | Tatanaa Sub Municipality |
|  | 3 | Pentecost Hospital | Social Welfare Sub Municipality |
|  |  |  |  |
| **ASHAIMAN POLYCLINIC** | 4 | Mother Of God Clinic | Mantseman |
|  | NA | St Mina Annex | Mantseman |
|  | 8 | General Family Hospital | Gbemi |
|  | 3 | Ashaiman Community Hospital | Gbemi |
|  | 4 | Trinity Community Hospital | Mantseman |
|  | 2 | St Mina Clinic | Blakpatsona |
|  | 1 | St Florence Clinic | Tsinai-agber |
|  | 1 | Ashiaman New Crystal Clinic | Tsinai-agber |
|  | 1 | Okanta Clinic | Maamomo |
|  | 1 | Darbem Clinic | Tsinai-agber |
|  | 4 | Community 22 Polyclinic | Niiman |
|  | 6 | General Family Hospital | Zenu |
|  | 5 | Lebanon Community Hospital | Zenu |
|  |  |  |  |
| **GREATER ACCRA REGIONAL HOSPITAL** | 4 | Bob Freman | Osu klottey |
|  | 7 | Nyaho Clinic | Osu klottey |
|  | 2 | Adabraka Polyclinic | Adabraka |
|  | 4 | Ghana Post Clinic | Osu |
|  | 3 | Civil Service Clinic | Tema Station |
|  | 4 | Cathederal Clinic | Osu Klottey |
|  | 3 | VRA Clinic | North Ridge |
|  | 3 | Tema Station Clinic | Tema Station |
|  |  |  |  |
| **LEKMA Hospital** | 2 | Manna Mission Hospital | Teshie North Sub-district |
|  | 5 | Family Health Hospital | Teshie South Sub-district |
|  | 5 | LEKMA Polyclinic | Nungua North |
|  |  |  |  |
| **SHAI OSU DOKU HOSPITAL** | 19 | St Andrews Catholic Hospital | Agomeda Sub-District |
|  | 60 | Osudoku Health Centre | Osudoku Sub District |
|  | 4 | Dangme West Hospital Complex | Dodowa Sub District |
|  | 13 | Valley View University Hospital | Oyibi |
|  |  |  |  |
| **TEMA GENERAL HOSPITAL** | 5 | Crocodile Matchete Clinic | Manhean |
|  | 1 | Bengali Clinic | Tema Central-South |
|  | 8 | Tema New Crystal Clinic | Tema Central-North |
|  | 8 | Manhean Health Centre | Manhean |
|  | 5 | GPHA Clinic | Community 2, 3 & 5 |
|  | 6 | Tema Polyclinic | Community 2, 3 & 5 |
|  | 30 | Dangme Community Hospital | Prampram Sub District |
|  | 34 | Omari Clinic | Afienya Mataheko sub-district |
|  | 35 | Old Ningo Health Centre | Old Ningo Sub-district |
|  | 24 | Prampram Polyclinic | Prampram Sub District |
|  | 11 | Speed Medical | Gbetsile |
|  | 6 | New Crystal Clinic (Kakasunaka Branch) | Gbetsile |
|  | 5 | All Care Medical Centre | Kpone |
|  | 5 | Nadela Hospital | Kpone |
|  | 12 | Kpone Health Centre | Kpone |
|  | 7 | Port Medical Centre | Community 1 |
|  | 3 | Narhbita Hospital | Tema North |
|  | 15 | Afienya Chps | Afienya Mataheko sub-district |
|  |  |  |  |
| **MAAMOBI GENERAL HOSPITAL** | 2 | Urban Aid Clinic | Maamobi East |
|  | 2 | Nima Government Hospital | Nima East |
|  | 2 | Sape Agbo | Accra New Town |
|  | NA | Clinix Diagnostic centre | Accra New Town |
|  | 3 | Mallam Atta Clinic | Accra New Town |
|  | NA | Anthon Mem. Hospital Annex | Kotobabi |
| **These distances were obtained using google maps.* ***NA*** *(Not Available) indicates cases where distances could not be generated by Google Maps****.*** | | | |
| **MAIN-TESTING FACILITIES:** |  |  |  |
| NATIONAL PUBLIC HEALTH AND REFERENCE LABORATORY  CENTRAL LABORATORY, KORLE BU TEACHING HOSPITAL  NOGUCHI MEMORIAL INSTITUTE FOR MEDICAL RESEARCH | | | |
|  |  |  |  |

| **NORTHERN REGION** | | | |
| --- | --- | --- | --- |
| **HUBS** | **Distance (Km)*** | **SPOKES** | **SUB-DISTRICT** |
| **SAVELUGU MUNICIPAL HOSPITAL** | 49 | Pishigu Health Centre | Pishigu |
|  | 68 | Karaga District Hospital | Karaga |
|  | 14 | Nanton Health Centre | Nanton |
|  |  |  |  |
| **YENDI MUNICIPAL HOSPITAL** | 63 | Gushiegu District Hospital | Gushiegu |
|  | 76 | Tatale Polyclinic | Tatale |
|  | 50 | Zabzugu District Hospital | Zabzugu |
|  | 39 | Bincheratanga Health Centre | Bincheratanga |
|  |  |  |  |
| **BIMBILLA MUNICIPAL HOSPITAL** | 36 | Nakpale Health Centre | Nakpale |
|  | 35 | Lungni Health Centre | Lungni |
|  | 46 | Kpandai District Hospital | Kpandai |
|  | 45 | ECG Hospital | Kpandai |
|  |  |  |  |
| **TAMALE PUBLIC HEALTH LABORATORY** | 3 | Bilpela Health Centre | Bilpeila |
|  | 4 | Kalpohin Health Centre | Taha |
|  | 3 | Tamale Central Hospital | Tamale Central |
|  | 2 | Tamale SDA Hospital | Tamale Central |
|  | 4 | Tamale West Hospital | Tamale Central |
|  | 10 | St Lucy Polyclinic | Choggu |
|  |  |  |  |
| **These distances were obtained using google maps* | | | |
| **MAIN TESTING FACILITY:** | | | |
| TAMALE PUBLIC HEALTH LABORATORY | | | |
