# Supplementary material for: Implementation of an Integrated Sample Referral System (ISRS) in Ghana: Successes and Lessons Learnt from a Pilot Study in the Northern and Greater Accra Regions
Source: PLOS Glob Public Health. 2025 Sep 11;5(9):e0004735. doi: 10.1371/journal.pgph.0004735 (PMC12425209; doi:10.1371/journal.pgph.0004735)
Supplement: S2 Appendix — (DOCX) [file pgph.0004735.s002.docx]

*VL – Viral load; EID – Early Infant Diagnosis; TB – Tuberculosis; YF – Yellow fever; AFP – Acute Flaccid Paralysis; RT – Room Temp; AT – Ambient Temp.*

*Possible factors for rejection: improper specimen transport leading to hemolysis; improper packaging; improper documentation; inadequate volume or overfilling of specimen container; incorrect specimen container/tube; specimen received in leaked, cracked or broken containers, specimen not appropriate for a particular test, etc.*

**Reviewed by:** ……………………………………………………... **Sign:** ……………..…………………………….. **Date:** ……………………
